# Retraction: 1, 25-D_3_ protects from cerebral ischemia by maintaining BBB permeability via PPAR-γ activation

**DOI:** 10.3389/fncel.2023.1355800

**Published:** 2023-12-21

**Authors:** Frontiers Editorial Office

The journal retracts the 17th December 2018 article cited above.

Following publication, concerns were raised regarding the integrity of the images in the published figures. The authors failed to provide a satisfactory explanation during the investigation, which was conducted in accordance with Frontiers' policies. As a result, the data and conclusions of the article have been deemed unreliable and the article has been retracted.

This retraction was approved by the Chief Editors of Frontiers in Cellular Neuroscience and the Chief Executive Editor of Frontiers. The authors did not agree to this retraction.

